# A comparative study on the chronic responses of titanium dioxide nanoparticles on aerobic granular sludge and algal–bacterial granular sludge processes

**DOI:** 10.1007/s11356-024-35581-z

**Published:** 2024-11-20

**Authors:** Alfonz Kedves, Henrik Haspel, Çağdaş Yavuz, Bence Kutus, Zoltán Kónya

**Affiliations:** 1 Department of Applied and Environmental Chemistry, University of Szeged, Szeged, Hungary; 2 HUN-REN Reaction Kinetics and Surface Chemistry Research Group, Szeged, Hungary; 3 Department of Molecular and Analytical Chemistry, University of Szeged, Szeged, Hungary

**Keywords:** Titanium dioxide nanoparticles, Aerobic granular sludge, Algal–bacterial granular sludge, Microbial activity, Chronic response

## Abstract

The chronic effects of titanium dioxide nanoparticles (TiO_2_ NPs) on aerobic granular sludge (AGS) and algal–bacterial granular sludge (ABGS) was examined in this study. Sequencing batch bioreactors (SBRs) and photo sequencing batch bioreactors (PSBRs) were operated with synthetic wastewater containing 0, 1, 5, 10, 20, 30, and 50 mg L^−1^ TiO_2_ NPs for 10 days. Nanoparticles at concentrations of 1 and 5 mg L^−1^ did not impact nutrient removal but led to an increase in extracellular polymeric substances (EPSs), primarily in protein (PN). With increasing nanoparticle concentration, the negative effect became more pronounced, mainly in the AGS SBRs. At 50 mg L^−1^ TiO_2_, chemical oxygen demand (COD), ammonia–nitrogen (NH_3_-N), and phosphorus (PO_4_^3−^) removal decreased by 20.9%, 12.2%, and 35.1% in AGS, respectively, while in ABGS, they reached only 13.4%, 5.7%, and 14.2%. ABGS exhibited steady-state nutrient removal at 30 and 50 mg L^−1^ TiO_2_ NPs after around 5 days. The higher microbial activity and EPS content in the sludge, coupled with the symbiotic relationship between algae and bacteria, contributed to the higher tolerance of ABGS to nanoparticles. Finally, although nanoparticles reduced biomass in both types of bioreactors, the accumulation of TiO_2_ NPs in the sludge, confirmed by Energy-dispersive X-ray spectroscopy analysis, and the absence of detectable titanium concentrations in the effluent wastewater, measured by Inductively-coupled plasma mass spectrometry, may be attributed to the specific operational conditions of this study, including the relatively short operation period (10 days) and high initial MLSS concentration (6 g L^−1^).

**Supplementary Information:**

The online version contains supplementary material available at 10.1007/s11356-024-35581-z.

## Introduction

Engineered nanoparticles (NPs) have found extensive applications due to their unique physical and chemical attributes in various fields including catalysts (Hou et al. 2019), electronics (Qamar et al. 2023), textiles (Rashid et al. 2021), and others. Titanium dioxide (TiO_2_) NPs, as a commercially significant and widely utilized nanoparticle, have gained prominence for their versatile applications such as in personal skincare products (Kumari and Virdi 2023), toothpastes (Al-Salman et al. 2020), and as a white pigment material (Mohammadparast and Mallard 2023) owing to their excellent stability and photocatalytic activity. TiO_2_ NPs have emerged as one of the most extensively manufactured nanomaterials (> 10,000 tons/year), it raises concerns regarding their potential environmental release, posing risks to microorganisms in the ecosystems (Kedves and Kónya 2024). Numerous studies have demonstrated the potential negative effects of these NPs on various organisms including algae (Natarajan et al. 2022), bacteria (Tahir et al. 2023), and fungi (Najibi Ilkhechi et al. 2021), underscoring the need for a comprehensive assessment and management of their environmental impact.

The widespread use of nanoparticles has led to their significant presence in wastewater. TiO_2_ nanoparticles were found both in sewage and sludge at concentrations up to 3 mg L^−1^ (Wang et al. 2017) and 23 mg kg^−1^ (Gottschalk et al. 2009), respectively. During biological wastewater treatment, TiO_2_ NPs could have numerous negative effect on bioreactors efficiency. Yuan et al. (2021) reported that the specific resistance to filtration of activated sludge (AS) increased after introducing 1 mg L^−1^ TiO_2_ NPs, while in another study the microbial diversity slightly shifted at 2 mg L^−1^ TiO_2_ NPs after 8 h (Cervantes-Avilés et al. 2021). In studies, where nanoparticle concentrations were increased to 5 or 10 mg L^−1^, either significant decrease in the removal rates of total nitrogen and phosphate after 6–8 days of exposure (Li et al. 2017; Zheng et al. 2011), or a decline in floc stability was observed (Zhou et al. 2019).

Mishima and Nakamura (1991) were the first to report on the aerobic granular sludge (AGS) wastewater treatment process, which has emerged as a promising technology for treating municipal and industrial waters. AGS offers advantages over activated sludge, such as greater stability, higher biomass content in the bioreactor, smaller footprint, and higher tolerance to toxic substances (Q. Jiang et al. 2022a, b; Zhou et al. 2022). Consequently, it has become one of the most extensively studied technologies in wastewater treatment over the past 30 years. Recently, algal–bacterial granular sludge (ABGS) has garnered attention due to its strong symbiotic relationship between algae and bacteria, resulting in excellent pollutant removal capabilities from wastewater (Fard and Wu 2023; Liu et al. 2022). Both AGS and ABGS have demonstrated the ability to simultaneously remove organic matter, phosphorus, and nitrogen from high-strength industrial wastewater (Li et al. 2022; Lochmatter et al. 2013), treat leachate (Ilmasari et al. 2023), and remove heavy metals (Kedves et al. 2024; Purba et al. 2020).

These two technologies are suitable for widespread applications due to the structure of the granules and the high amount of extracellular polymeric substances (EPS) they contain. EPS consists of two main components: polysaccharides and proteins. While the former predominantly forms the outer part of the granules, the proteins typically constitute the main component of the granules’ inner layer (Nuramkhaan et al. 2019; Samaei et al. 2023). Since AGS technology is already used on an industrial scale for the treatment of industrial and municipal wastewater (Hamza et al. 2022), several studies examined the impact of the increasingly produced nanoparticles on the AGS wastewater treatment.

In wastewater treatment systems, mixed liquor suspended solids (MLSS) are directly related to the system resilience against contaminants (Wang et al. 2023). Accordingly, where possible, the amount of nanoparticles introduced in the various experiments was also calculated based on MLSS. Quan et al. (2015) observed a decrease in biomass and in microbial activity during the long-term exposure of silver NPs of 5 and 50 mg L^−1^ (1.67 and 16.67 mg gMLSS^−1^). In another studies, the increase of cupric oxide (CuO) NPs concentration from 5 to 50 mg L^−1^ (1.67 to 16.67 mg gMLSS^−1^) significantly reduced the removal of phosphorus (Zheng et al. 2017), while Cu NPs at 5 mg L^−1^ (0.38 mg gMLSS^−1^) inhibited the nitrogen removal capacity by 51.9% during long-term exposure (Cheng et al. 2019a, b, c). The shock loading of zinc oxide (ZnO) NPs at 1–100 mg L^−1^ (0.23–22.73 mg gMLSS^−1^) caused acute toxic effect on microbial activity (He et al. 2017b), whilst the nitrification and denitrification processes were inhibited even at 20 mg L^−1^ in the long term (He et al. 2017a). Moreover, nanoparticles tend to enrich in the sludge, as Xiao et al. (2024) found the 95% of ZnO NPs in the sludge when the wastewater contained 10 mg L^−1^ NPs. Another study showed no harmful effects of ZnO NPs up to 1 mg L^−1^ (0.17 mg gMLSS^−1^), whereas the ammonia and phosphorus removal significantly decreased at 10 mg L^−1^ (1.67 mg gMLSS^−1^) (Xiao et al. 2022). So far, only one study examined the effect of TiO_2_ NPs on AGS at a single concentration of 50 mg L^−1^ (Y. Jiang et al. 2022a, b), while in the case of ABGS, the effect of TiO_2_ NPs on granule formation was studied (B. Li et al. 2015a, b). Considering that i) previous studies covered the effects of Zn and Cu-based NPs, ii) TiO_2_ NPs are present in wastewater, iii) AGS is used on full-scale, and iv) the future industrial-scale utilization of ABGS is highly likely, the investigation of the effects of TiO_2_ NPs on AGS and ABGS became vital.

Herein, we report the chronic impact (over 10 days) of TiO_2_ NPs at a series of concentrations (0, 1, 5, 10, 20, 30, and 50 mg L^−1^, corresponding to 0.17, 0.83, 1.65, 3.31, 4.96, and 8.26 mg gMLSS^−1^) on the nutrient removal efficiency, the microbial activity, and the extracellular polymeric substances of both, the AGS and ABGS processes. These findings provide fundamental insights into the TiO_2_ nanoparticle tolerance on aerobic granular and algal–bacterial aerobic granular sludge, and thus are expected to contribute to the knowledge on the operation of AGS and ABGS in treating TiO_2_ NPs contaminated wastewater.

## Materials and methods

### Experimental

All bioreactors were inoculated with aerobic granular sludge and algal–bacterial granular sludge freshly collected from mother reactors operated for over half a year in our laboratory. The AGS sequencing batch reactors (SBRs) and ABGS photo-sequencing batch reactors (PSBRs) were continuously fed with synthetic wastewater (SWW) (Table S1) containing 1, 5, 10, 20, 30, and 50 mg L^−1^ TiO_2_ NPs, every four hours for 10 days. The hydraulic retention time of SWW in the bioreactors was 8 h with an effective volume of 1.4 L. The average mixed liquor suspended solids and sludge volume index (MLSS and SVI_5_) were 6.05 ± 0.2 g L^−1^ and 24.6 ± 0.1 mL g^−1^, respectively. Further details on the components of the bioreactors, the configuration of SBR and PSBR, and the constituents of SWW are provided in the *Supplementary Information*. TiO_2_ nanoparticles were synthesized through a modified nonaqueous solvothermal process (Z. Q. Li et al. 2015a, b), and were characterized by using a Rigaku Miniflex-II X-ray diffractometer (Fig. S1*a*), a Bruker Vertex 70 FT-IR instrument (Fig. S1*b*), and a Hitachi S-4700 Type II scanning electron microscope (SEM) with 10 kV accelerating voltage equipped (Fig. S1*d*) with a Röntec QX2 energy dispersive X-ray spectrometer (EDX) (Fig. S1*c,* see *Supporting Information*).

### Analysis of effluent water, sludge properties, and microbial activity

Every 12 h over a period of 10 days, the concentration of chemical oxygen demand (COD), phosphorus (PO_4_^3−^), nitrate-nitrogen (NO_3_-N), nitrite-nitrogen (NO_2_-N), and ammonia nitrogen (NH_3_-N) in the effluent of the SBRs and PSBRs was measured using Hanna kits (HI93754B-25, HI93717-01, HI93728-01, HI93708-01, and HI93715-01) with a spectrophotometer HI83399. Additionally, titanium content in the effluent wastewater was measured via Inductively-coupled plasma mass spectrometry (ICP-MS) using an Agilent 7900 instrument with 15.0 L min^−1^ Ar carrier gas. Samples were filtered through a 0.45 μm syringe filter and stored at − 4 °C, and prior to analysis cc. HNO_3_ (NORMATOM by VWR Chemicals, final concentration: 1 wt%) and solutions of the internal standards ^45^Sc and ^89^Y (ARISTAR by VWR Chemicals, final concentration: 100 ppb) were added. Calibration was performed between 0 and 50 ppb Ti using the same procedure, signals of the ^47^Ti, ^48^Ti, and ^49^Ti isotopes were monitored with and without using He cell collision mode.

On the tenth day of the experiments, the MLSS, SVI_5_, and EPS content of both granular sludges were determined. Additionally, to assess the impact of TiO_2_ NPs on the microbial activity, specific phosphorus uptake rate (SPUR), specific ammonia, nitrite, and nitrate uptake rates (SAUR, SNIUR, and SNUR) were determined. Finally, the effect of TiO_2_ NPs on the structure of the aerobic and algal–bacterial granular sludge was examined using a Hitachi S-4700 Type II scanning electron microscope (SEM) and EDX. Detailed descriptions of the MLSS, SVI_5_, EPS, SPUR, SAUR, SNIUR, and SNUR measurements, as well as the preparation of sludge samples for SEM investigation, can be found in the *Supplementary Information*.

## Results and discussion

### Sludge properties and EPS production in AGS and ABGS

**Fig. 1:**
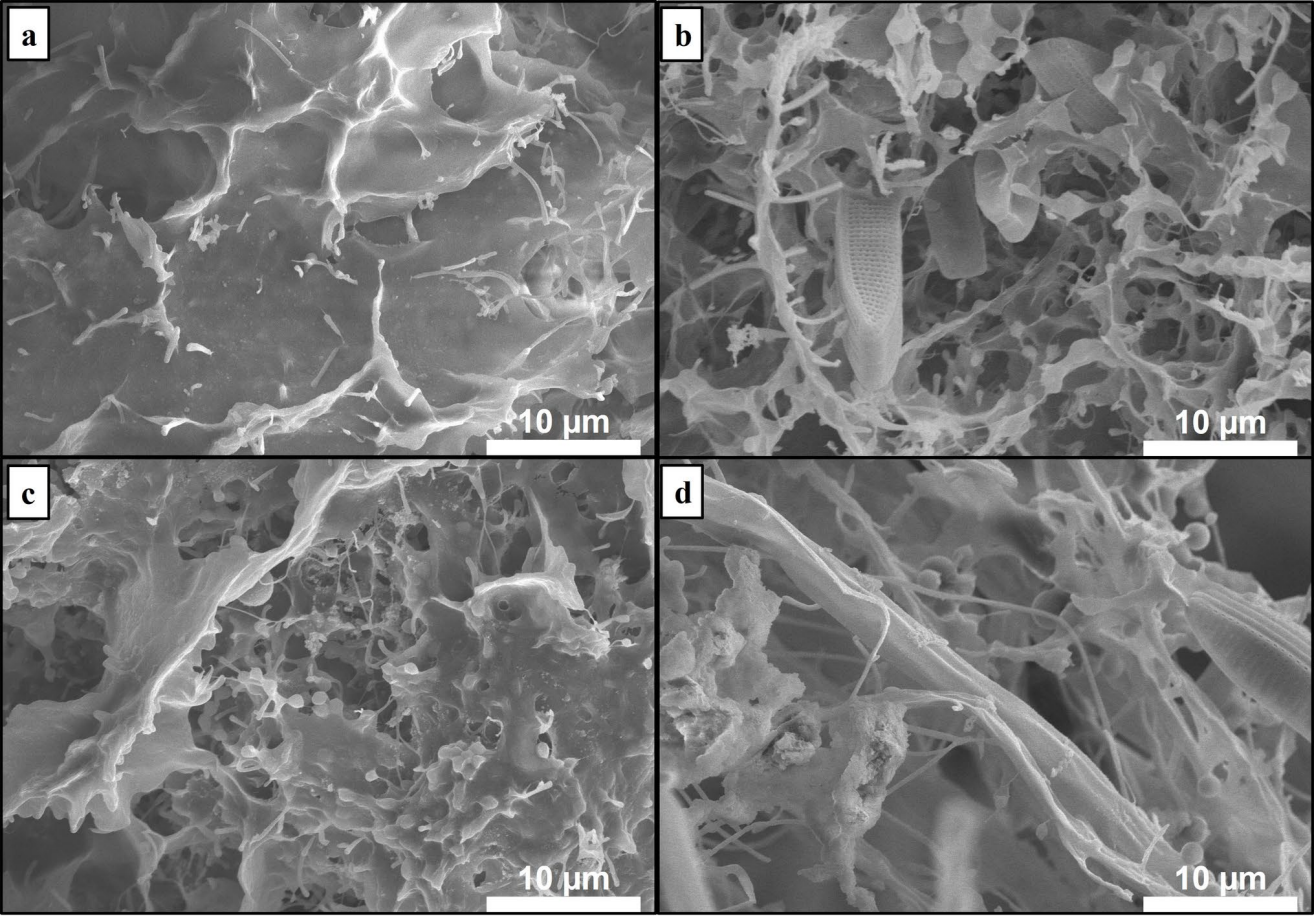
Scanning electron microscope (SEM) images of the investigated sludges. The surface of the initial **a)** aerobic granular sludge (AGS), and the **b)** algal–bacterial granular sludge (ABGS). The exterior of the **c)** AGS, and the **d)** ABGS after the introduction of 50 mg L^−1^ TiO_2_ NPs

Compared to activated sludge (AS), both AGS SBRs and ABGS PSBRs contain a significant amount of biomass, along with high levels of extracellular polymeric substances (EPSs), primarily consisting of protein (PN) and polysaccharide (PS). EPS not only protect microorganisms from harmful substances, such as nanoparticles, but also contribute to good settling ability and possess biosorption properties (Hakim et al. 2023; Zheng et al. 2019). At the beginning of each experiment, the MLSS and SVI_5_ of the sludges were approximately 6.05 ± 0.2 g L^−1^ and 24.6 ± 0.1 mL g^−1^, while the EPS content in AGS and ABGS was 106.5 ± 6.1 and 129.6 ± 6.7 mg g^−1^ MLVSS, respectively. As shown in Fig. 1a, numerous microorganisms were embedded in the polymer matrix on the surface of the aerobic granules, whereas algae can be seen alongside other microorganisms within the exterior of the algal–bacterial sludge (Fig. 1b) (Salimon et al. 2020). Based on the sludge EDX analysis, the main differences between the types of sludge are nitrogen and phosphorus contents. In AGS 8.78 and 0.58 at%, in ABGS 9.14 and 0.88 at% nitrogen and phosphorus were found, respectively (Fig. S2). The difference originates from the presence of algae, as these microorganisms are capable of accumulating high amounts of these elements (Kube et al. 2018).

**Fig. 2:**
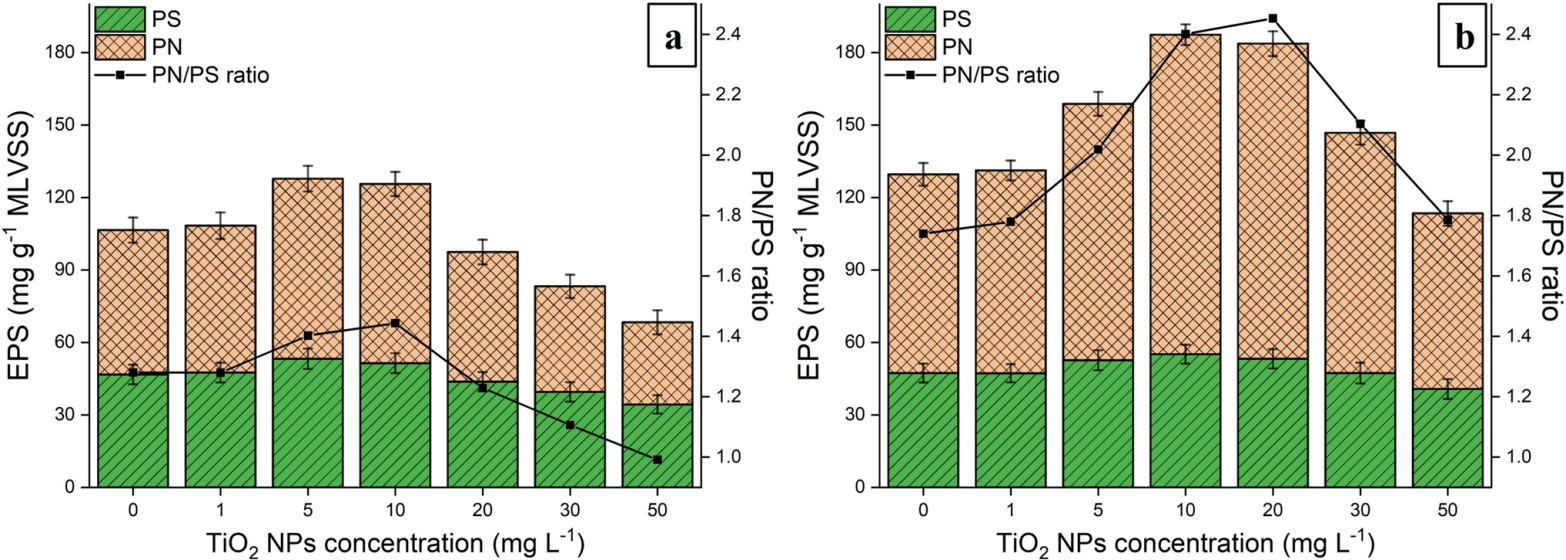
EPS (PN + PS) secretion in AGS and ABGS processes after addition of TiO_2_ NPs. EPS volume in **a)** AGS and **b)** ABGS after addition of TiO_2_ NPs

TiO_2_ NPs (with spherical morphology of a diameters ranging between 30 and 130 nm, see Fig. S1) at concentrations of 5 and 10 mg L^−1^ led to an increase in EPS in both granular sludges. This increase was accompanied by the rise in the PN/PS ratio, attributed to a higher volume of PN secretion after 10 days of exposure (Fig. 2). At 10 mg L^−1^ NPs, the PN content increased by 24% from 59.8 ± 5.1 to 74.2 ± 5 mg g^−1^ MLVSS, and by 60% from 82.3 ± 4.7 to 132.3 ± 4.3 mg g^−1^ MLVSS in AGS and ABGS, respectively. These results suggest that ABGS has a higher tolerance to the negative effects of nanoparticles compared to AGS, as it exhibited a greater capacity for protein secretion. In two previous studies, He et al. (2017a, b) and Xiao et al. (2022) investigated the chronic effects of zinc oxide nanoparticles on AGS and ABGS. During the experiments, it was observed that ZnO NPs caused a reduction in the amount of sludge EPS at concentrations as low as 10 mg L^−1^, significantly impacting the efficiency of the reactors. In our case, however, following the administration of 10 mg L^−1^ of TiO_2_, the quantity of polymer materials was higher than in the initial sludge. The low toxicity promoted bacterial production of polymer materials in the sludge, whereas higher toxicity reduced its quantity (Quan et al. 2015). We, therefore, presume that zinc oxide nanoparticles may be more toxic to the granular sludge.

The EPS content declined, and sludge properties changed with increasing nanoparticle content (20, 30, and 50 mg L^−1^ TiO_2_ NPs) in AGS. The polymer volume decreased to 97.4, 83.2, and 68.3 mg g^−1^ MLVSS, and the PN/PS ratio dropped to 1.22, 1.1, and 0.99, below those of the initial sludge (Fig. 2a). Simultaneously, the MLSS decreased to 5.96, 5.52, and 4.83 g L^−1^, while significant changes were not observed in the SVI_5_. This data suggests that the persistent presence of TiO_2_ NPs in the influent led to a decrease in biomass production in the AGS reactor. The long-term presence of low concentration (5 and 10 mg L^−1^) CuO NPs had a similar effect on AGS, i.e., the secretion of EPS was promoted, while a decrease in biomass and EPS content was observed at higher concentrations (50 mg L^−1^) (Zheng et al. 2017). The decreasing amount of biomass can be explained by the decrease in the size of the granules, since their average diameter declined from 600 to 350 µm at 50 mg L^−1^ NPs after 10 days. Along with the size reduction, the external structure of AGS also changed (Fig. 1c), as the polymer layer disappeared and rod-shaped microorganisms can be seen. The latter suggests a significant change in the microbial structure.

**Fig. 3:**
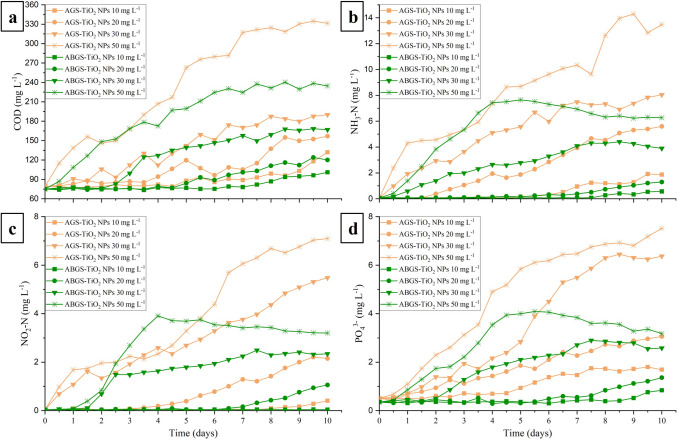
Influence of titanium dioxide nanoparticles (TiO_2_ NPs) on the removal of nutrients. **a** COD, **b** NH_3_-N, **c** NO_2_-N, and **d)** PO_4_^3−^ contents

The addition of TiO_2_ NPs at 20, 30, and 50 mg L^−1^ had distinct effects in PSBRs and SBRs. Although significant changes in sludge properties were not observed until the day 10 upon the addition of 20 and 30 mg L^−1^ NPs, the PN content remained higher compared to that of the initial ABGS with a PN/PS ratio of 2.45 and 2.11, respectively. In contrast, the PN and PS decreased from 82.3 ± 4.7 and 47.3 ± 3.9 mg g^−1^ MLVSS to 72.7 ± 5.1 and 40.7 ± 4.1 mg g^−1^ MLVSS at 50 mg L^−1^ TiO_2_ NPs, respectively (Fig. 3b). Simultaneously, the MLSS decreased to 3.69 ± 0.5 g L^−1^, and the SVI_5_ increased to 165.9 ± 7.6 mL g^−1^. The significant change occurred because filamentous microorganisms appeared on the outer part of the granules (Fig. 1d), leading to a loose structure with poorer settling ability and an increase in the granular sludge size from 0.6 mm to 2–8 mm.

### Performance on nutrients removal of AGS and ABGS

In order to characterize the efficiency of a biological wastewater treatment, the removal of organic matter, nitrogen, and phosphate need to be measured. The performance of AGS and ABGS bioreactors was assessed by recording the COD, NH_3_-N, NO_2_-N, NO_3_-N, and PO_4_^3−^ every third cycle (every 12 h) in each experiment over 10 days (Fig. 3). Although the removal of nutrients remained stable after introducing the nanoparticles at concentrations as low as 1 and 5 mg L^−1^, increasing NP content resulted in a gradually decreasing removal efficiency.

Upon the introduction of 10 and 20 mg L^−1^ nanoparticles into the AGS-SBR, the COD in the effluent began to increase after 5 and 2 days, and rising from 79 ± 2.1 to 132 ± 4.2 mg L^−1^, and 156.9 ± 3.7 mg L^−1^ on day 10, respectively (Fig. 3a). In contrast, in the ABGS PSBRs, the COD started to increase after 7 and 5 days with levels changing from 75 ± 1.4 to 101 ± 1.9 mg L^−1^, and 120 ± 1.7 mg L^−1^ by day 10, respectively. At concentration of 50 mg L^−1^, the COD removal rate declined after just half a day due to the shock loads of titanium. In the AGS, a continuous increase in COD was measured in the effluent reaching 331.5 ± 4.9 mg L^−1^ by day 10, while in the ABGS, COD removal reached a steady state after 6 days. Overall, the observations indicated that TiO_2_ nanoparticles had a lower impact on the heterotrophic microbial community in algal–bacterial sludge compared to other studies. In previous research, where the long-term impact of TiO_2_ nanoparticles on activated sludge was investigated, a decline in COD removal was already observed at concentrations as low as 1 or 2 mg L^−1^ (Cervantes-Avilés et al. 2021; Li et al. 2019). This suggests that heterotrophic microorganisms in AGS and ABGS are more tolerant to titanium dioxide nanoparticles, likely due to their robust structure and high EPS content.

Here, we observed a decrease in NH_3_-N removal after 5–2.5, and 8.5–7.5 days upon the exposure of the AGS and ABGS by 10 and 20 mg L^−1^ NP, respectively. The further increase in the TiO_2_ NPs content (30–50 mg L^−1^) caused significant inhibition of the aerobic sludge within half a day, with removal efficiency decreasing from 99.94% to 92.87%, and 88.33% by day 10 (Fig. 3b). The negative impact of nanoparticles on ammonia removal was less pronounced in ABGS. Although efficiency declined after the first day at 30 and 50 mg L^−1^ NPs, levels stabilized around days 6 and 5 with the removal rates reaching 96.31% and 94.26% by day 10, respectively.

The NO_2_-N contents in the effluent (Fig. 3c) exhibited similar variations to ammonia–nitrogen, suggesting that nanoparticles had a negative effect on both aerobic ammonia and nitrite oxidizing microorganisms. These microbes are predominantly located in the outer part of the granules, allowing TiO_2_ NPs to potentially attach to their surface and inhibit their microbial activity. Zheng et al. (2011) also observed a decreased removal rate of both ammonia and nitrite in activated sludge exposed to 50 mg L^−1^ TiO_2_ NPs, along with a drastically dropping nitrogen elimination to 24.4%. Throughout the experiments, the NO_3_-N content in the effluent remained constant in the algal–bacterial sludge. In the AGS, on the other hand, it increased to 10.2 and 36.7 mg L^−1^ as a response to the introduction of 30 and 50 mg L^−1^ TiO_2_ NPs, respectively.

When considering phosphorus removal, notable distinctions between AGS and ABGS are evident (Fig. 3d). At 10 and 20 mg L^−1^ NPs, phosphorus removal in ABGS began to decrease after 9 and 6 days with effluent PO_4_^3−^ contents of 0.84 ± 0.05 and 1.36 ± 0.12 mg L^−1^ after 10 days, respectively. However, in AGS, the efficiency declined after 3 and 2 days, resulting in PO_4_^3−^ amounts of 1.69 ± 0.8 and 3.06 ± 0.16 mg L^−1^ at day 10, respectively. Additionally, when titanium content of the influent wastewater was 30 and 50 mg L^−1^, the PO_4_^3−^ content in the effluent stabilized after 7 and 6 days in ABGS, respectively. By day 10, however, the phosphate concentration was similar or lower than that in AGS in response to 20 mg L^−1^ TiO_2_ NPs. Upon further increasing the TiO_2_ concentration, the phosphorus content increased from 0.51 ± 0.05 to 7.52 ± 0.39 mg L^−1^ in the latter along with a drop in the removal rate to 62.4%. In contrast, phosphorus concentration increased from 0.35 ± 0.05 to 3.18 ± 0.13 mg L^−1^, leading to a reduction in removal efficiency to 84.1% in ABGS. Li et al. (2017) also observed a decrease in phosphorus removal efficiency in AS, where it reduced from 89 to 68%, suggesting that granular sludges exhibit significantly higher resistance against titanium-dioxide.

A correlation between the PO_4_^3−^, and the NH_3_-N and NO_2_-N nitrogen removal results was found. The correlation is partly due to the fact that microorganisms that are sensitive to higher concentrations of ammonia and nitrite in the wastewater are also the ones capable of accumulating and removing phosphate, as previously observed (Zheng et al. 2017). The better nutrient removal efficiency of ABGS may also originate from the algae's high tolerance to heavy metals and their ability to remove nutrients from wastewater (Nguyen et al. 2022; Priyadarshini et al. 2019). In AGS, chronic exposure to titanium dioxide nanoparticles led to a more pronounced decrease in nutrient removal efficiency, resulting in a lower biomass quantity compared to that in the initial sludge after 10 days. This reduction in biomass could have potentially contributed to the release of nutrients from the polymer matrix, exacerbating the effluent's COD, nitrogen, and phosphorus content. Continuous monitoring of the titanium content in the effluent wastewater was conducted throughout the experiments, whose presence could not be detected. This in turn highlights the importance of preventing the pollutant release into the environment, if it accumulates in the sludge. The titanium content in AGS and ABGS was 8.25 and 2.66 at% at 50 mg L^−1^ TiO_2_ NP concentration (Fig. S3). The difference is likely due to the low penetration depth of the EDX sludge analysis. Since numerous filamentous microorganisms covered the surface of the granules in ABGS (Fig. 1d), only part of the elemental information comes from the Ti–rich granules, which in turn results in a lower measured titanium content.

### Influence of TiO_2_ NPs on the microbial activity of AGS and ABGS

**Fig. 4:**
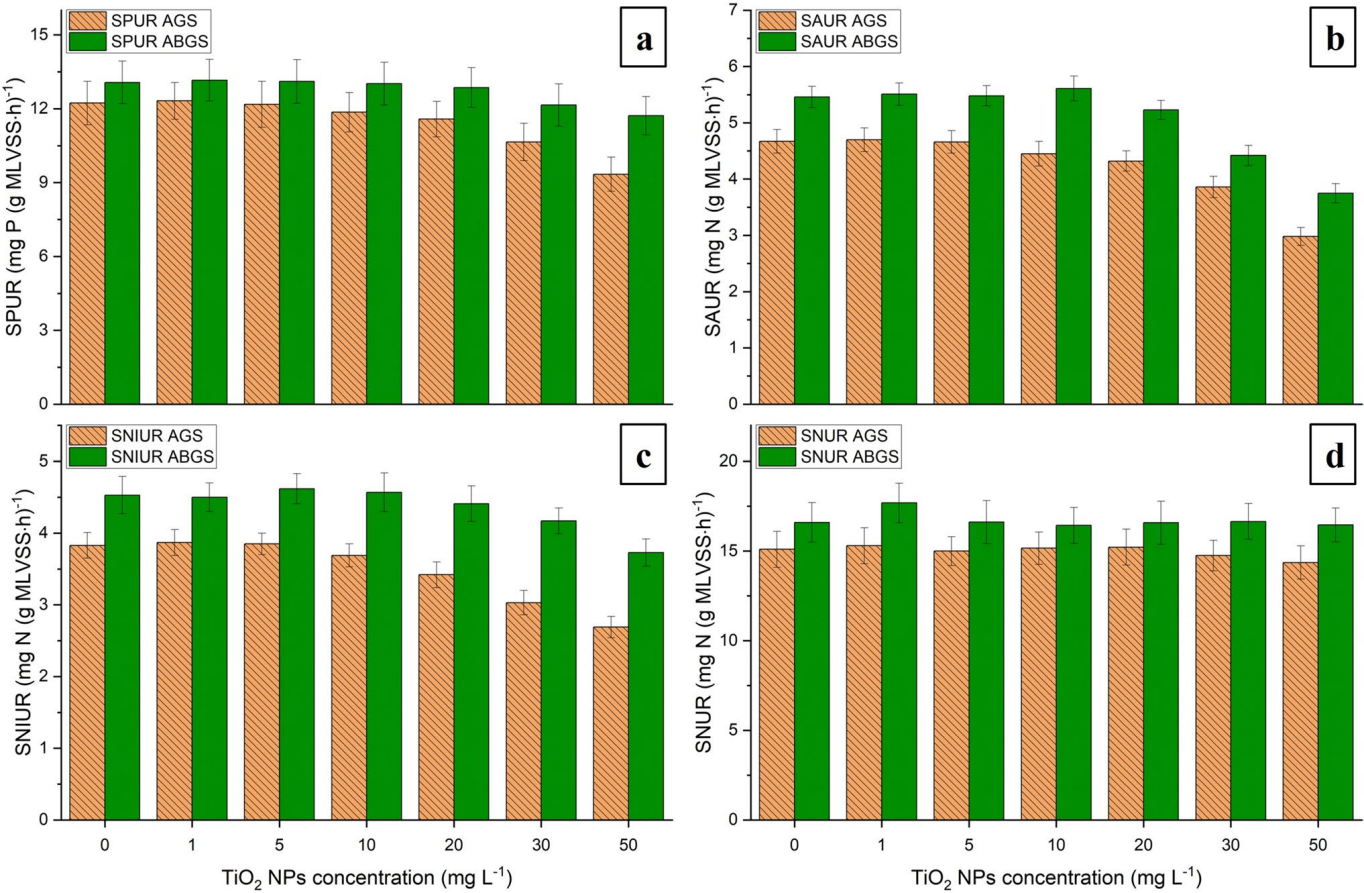
Influence of titanium dioxide nanoparticles (TiO_2_ NPs) on the microbial activities of aerobic granular sludge (AGS) and algal–bacterial granular sludge (ABGS). **a** specific phosphorus uptake rate (SPUR), **b** specific ammonia uptake rate (SAUR), **c** specific nitrite uptake rate (SNIUR), and **d)** specific nitrate uptake rate (SNUR)

As shown in Fig. 4a, the specific phosphorus uptake rate (SPUR) of AGS remained unaffected after the addition of TiO_2_ NPs at 1 and 5 mg L^−1^. However, with further increase the concentration to 10, 20, 30, and 50 mg L^−1^, the SPUR decreased from 12.23 to 11.86, 11.58, 10.65, and 9.34 mg P (g MLVSS∙h) ^−1^, respectively. Similar results were observed in previous studies, where increasing Ag, ZnO, and CeO_2_ NPs concentrations in AGS resulted in a significant reduction in phosphorus uptake, leading to an increased phosphate volume in the effluent water (Wang et al. 2016b, 2016a; Xu et al. 2017).

Unlike AGS, the phosphorus uptake of ABGS remained stable at ≤ 20 mg L^−1^ NPs, and then declining from 13.07 to 12.15 and 11.72 mg P/(g MLVSS∙h) ^−1^ after the addition of 30 and 50 mg L^−1^ nanoparticles. These results coincide with the trend found in phosphorus removal, where the PO_4_^3−^ amount in effluent was consistently lower in ABGS, than in AGS at the same contaminant concentration. This indicates that TiO_2_ had a greater impact on SPUR in aerobic sludge, and on the microbial community in ABGS. This in turn plays an important role in phosphorus removal and exhibited a better tolerance against the nanoparticles. The smaller change in SPUR can be attributed to i) their higher resilience against metal contaminants (Xiao et al. 2023), and ii) the better phosphorus uptake capacity of algae (Boelee et al. 2011). At concentrations of ≥ 20 mg L^−1^ TiO_2_ NPs, both AGS and ABGS exhibited a major drop in SAUR. The microbial activity declined by 7.49%, 17.34%, and 36.18% in AGS, and 4.21%, 19.04%, and 31.31% in ABGS. Interestingly, despite the significant decrease in microbial activity in the algal–bacterial sludge, it showed a higher ammonia removal compared to that in AGS as evidenced by the higher ammonia uptake value in ABGS (Fig. 4b). Quan et al. (2015) observed a similar significant reduction in SAUR in AGS, where the ammonia uptake dropped by 28% along with a 50 mg L^−1^ Ag NPs concentration in the effluent.

A similar trend could be observed in the case of SNIUR (Fig. 4c). The nitrite uptake of AGS decreased by 10.70%, 20.89%, and 29.76%, while in ABGS, it dropped by 2.65%, 7.95%, and 17.66% after the introduction of TiO_2_ NPs at concentrations of 20, 30, and 50 mg L^−1^, respectively. The changes were lower in the SNIUR of the algal–bacterial sludge, resulting in lower NO_2_-N content in the effluent. These findings are consistent with prior reports, wherein an increase in ammonia and nitrite contents were found in the effluent, when the SAUR and SNIUR were lower compared to the initial granular sludge (Wang et al. 2016b, 2015). Since nitrite-oxidizing microorganisms are typically found in the outer layers of the granules, the decrease in SNIUR can be lower in ABGS than in AGS. This can be due to i) the higher amount of EPS (Fig. 2) and ii) the filamentous microorganisms, which increase the surface area and reduce the nanoparticle exposure of the initial granules.

The SNUR in the initial ABGS was 16.6 mg N/(g MLVSS∙h) ^−1^ and remained unchanged during the experiments. This finding indicates that nitrate removal in algal–bacterial sludge remained constant, suggesting that the elevated titanium dioxide content did not hinder the microbial activity of denitrifying bacteria. The nitrate uptake in the control AGS was 15.1 mg N/(g MLVSS∙h) ^−1^, and no significant change was observed at TiO_2_ NP concentrations ≤ 20 mg L^−1^. However, when the nanoparticle concentration was increased to 30 and 50 mg L^−1^, the SNUR in AGS declined to 14.85 and 14.53 N/(g MLVSS∙h) ^−1^, respectively. This observation is consistent with the elevated nitrate level in the effluent and suggests that the nanoparticles were able to penetrate the interior of the granules, thereby reducing the microbial activity of the denitrifying anaerobic microorganisms. This reduction may have occurred due to a significant decrease in the quantity of extracellular polymeric substances (Fig. 2a), which normally protect the cells.

### Possible mechanisms

(i) at TiO_2_ NP concentrations of 1 and 5 mg L^−1^, no negative effects were observed on the microbial activity of AGS, while the EPS content increased and nutrient removal efficiency did not decrease. In contrast, both the microbial activity and EPS amount of ABGS increased even after the addition of 10 mg L^−1^ TiO_2_ NPs, and nutrient removal remained stable. This slight "positive" effect can be attributed to the fact that low amounts of heavy metals can stimulate the enzymatic activity of microorganisms and their EPS secretion (Cheng et al. 2019a, b, c), while the extended tolerance threshold of ABGS may be due to the tolerance of algae to heavy metals.
(ii) based on the ICP measurements, TiO_2_ NPs exhibited a poor ability to release Ti^1+^, with only 2% being released after 4 h and just 5% after 24 h. This small amount of Ti^1+^ and TiO_2_ NPs could penetrate through the EPS and infiltrate the microorganisms, altering their selective permeability and leading to cell death (Kedves and Kónya 2024). However, the released ions were unable to penetrate the granular sludge, resulting in stable nitrate removal in both types of sludge.
(iii) EPS serve as the initial protective barrier for microbial aggregates, enabling them to come into direct contact with and adsorb metal ions and nanoparticles present in wastewater (Cheng et al. 2019a, b, c). When TiO_2_ NPs and Ti^1+^ attached to the surface or penetrated the interior of the granules at a tolerable amount (in AGS it was 5–10 mg L^−1^, while in ABGS 10–20 mg L^−1^ TiO_2_ NPs), the cells responded by producing more EPS, which increased by 18% in AGS and by 41% in ABGS, as a protective mechanism to prevent entry and mitigate excessive oxidative stress.
(iv) the response of microbial activity and EPS production differed between ABGS and AGS. The algal–bacterial consortia in ABGS secreted a higher amount of EPS to mitigate the harmful effects of TiO_2_ NPs, while AGS produced less EPS. Consequently, microbial activity and nutrient removal efficiency remained higher in ABGS. This EPS-driven defense mechanism may be linked to the energy dynamics of ABGS, where the symbiotic relationship between algae and bacteria promotes the secretion of more polymers for protection against NPs. In contrast, AGS may direct additional energy toward detoxification or cellular repair processes, such as actively expelling excess NPs or ions from sensitive cell areas (Zhang et al. 2018).
(v) the contact between TiO_2_ NPs and the microalgae in the outer layer of the granules caused limited light availability in case of ABGS. This reduction in light exposure diminished the viability of algae viability, subsequently leading to decreased nitrogen and phosphorus absorption (Xiao et al. 2022). Furthermore, the proliferation of filamentous microorganisms on the surface of the sludge, following the addition of 50 mg L^−1^ TiO_2_ NPs, could also cause a shading effect on the algae.

## Conclusions

Titanium dioxide (TiO_2_) nanoparticles (NPs) are widely utilized in versatile applications in various fields of our everyday life, which makes them one of the most extensively manufactured nanomaterials. Still, their potential environmental effect on the microorganisms in biological wastewater treatments has not been examined before. In this study, the chronic effects of TiO_2_ NPs on aerobic granular sludge (AGS) and algal–bacterial aerobic granular sludge (ABGS) biological wastewater treatment processes were investigated over a 10-day period. At low nanoparticle concentrations (1 and 5 mg L^−1^), the performance of the bioreactors remained unaffected, whereas at higher concentrations (10, 20, 30, and 50 mg L^−1^) a decreased nutrient removal efficiency was found. ABGS exhibited higher tolerance to elevated nanoparticle concentrations than that found in AGS, hence maintaining a more stable treatment efficiency and microbial activity. Some minor changes were observed in the algal–bacterial sludge structure, characterized by filamentous microorganisms surrounding the granules and reducing its settling ability. In contrast, TiO_2_ NPs have a more pronounced AGS showed negative effects at a concentration of 10 mg L^−1^, with decreasing efficiency in nutrient removal, microbial activity, and EPS quantity compared to ABGS. These findings suggest a strong symbiotic relationship between algae and bacteria in the algal–bacterial granular sludge, enabling the development of a more effective defense mechanism against nanoparticles. Despite the reduction in performance observed in both bioreactors, nanoparticles were not detectable in the effluent wastewater, as they accumulated in the sludge.

## Supplementary Information

Below is the link to the electronic supplementary material.

Supplementary file1 (DOCX 666 KB)

## Data Availability

The authors confirm that the data supporting the findings of this study are available within the article and extra information can be obtained by emailing the corresponding author, upon reasonable request.
