# Supplementary material for: A comparative study on the chronic responses of titanium dioxide nanoparticles on aerobic granular sludge and algal–bacterial granular sludge processes
Source: Environ Sci Pollut Res Int. 2024 Nov 20;32(54):30159–69. doi: 10.1007/s11356-024-35581-z (PMC12804245; doi:10.1007/s11356-024-35581-z)
Supplement: Supplementary file 1 — Supplementary file1 (DOCX 666 KB) [file 11356_2024_35581_MOESM1_ESM.docx]

**A comparative study on the chronic responses of titanium dioxide nanoparticles on aerobic granular sludge and algal-bacterial granular sludge processes**

**Environmental Science and Pollution Research**

Alfonz Kedves^1^, Henrik Haspel^1,2^, Çağdaş Yavuz^1^, Bence Kutus^3^, Zoltán Kónya^1,2^

*^1^Department of Applied and Environmental Chemistry, University of Szeged, Szeged, Hungary.*

*^2^HUN-REN Reaction Kinetics and Surface Chemistry Research Group, Szeged, Hungary.*

*^3^Department of Molecular and Analytical Chemistry, University of Szeged, Szeged, Hungary.*

Corresponding author: Alfonz Kedves, PhD, Department of Applied and Environmental Chemistry, University of Szeged, H-6720 Szeged, Rerrich Béla tér 1, Hungary. E-mail address: [kedvesalfonz@chem.u-szeged.hu](mailto:kedvesalfonz@chem.u-szeged.hu)

**2. Material and methods**

**2.1. Configuration of AGS and ABGS bioreactors**

Urban wastewater treatment plant (Szeged, Hungary) provided the activated sludge (AS). Aerobic granular sludge (AGS) was cultivated in two identical sequencing batch reactors (SBRs) with a height-to-diameter ratio of 7 and an effective working volume of 1.4 L. An artificial light source delivered 12-hour daily illumination near the algal-bacterial granular sludge (ABGS) SBR at an intensity of approximately 6500 lux, while the AGS reactor remained covered by a hood to prevent light exposure. To ensure oxygenation and complete mixing, air bubbles (superficial air velocity of 2 cm s^-1^) were supplied by diffusers located at the reactor's bottom. Each cycle involved a decant/feed volume of 50% of the total volume, with an 8 h hydraulic retention time. The bioreactor operated at 21 ± 3 °C in 4-hour cycles, encompassing filling (5 min), aeration (225 min), settling (5 min), and withdrawal (5 min) periods. Peristaltic pumps controlled the filling and withdrawal of wastewater at specified flow rates. Characteristics of the synthetic wastewater (SWW) are summarized in *Table S1*.

**Table S1** - Characteristics of synthetic wastewater and Sims South Wastewater Treatment activated sludge mixture

| **Parameters** | **Synthetic wastewater** |
| --- | --- |
| pH | 7.3 ± 0.7 |
| Ammonia – nitrogen (NH_3_ – N) (mg L^-1^) | 110 ± 1 |
| Phosphorus (PO_4_^3-^) (mg L^-1^) | 20 ± 1 |
| Chemical oxygen demand (COD) (mg L^-1^) | 1200 ± 9.67 |
| Sodium bicarbonate (NaHCO_3_) (mg L^-1^) | 250 ± 2 |
| Calcium chloride (CaCl_2_) (mg L^-1^) | 30 ± 0.5 |
| Magnesium sulphate (MgSO_4_) (mg L^-1^) | 50 ± 0.7 |
| Trace element solution | (Kedves et al., 2020) |

**2.2. Preparation and characterization of TiO_2_ NPs**

All chemicals were used as received without any further purifications. Based on the previously published technique, TiO_2_ nanospheres were synthesized through a modified nonaqueous solvothermal process. Titanium isopropoxide (TIP, 5 mL) was dissolved in 130 mL of anhydrous acetone, and after 20 minutes of stirring at room temperature, the solution was taken to a 250 mL Teflon-lined stainless steel autoclave and kept at 180 °C for 10 hours (Li et al., 2015). The yellowish sediment was gathered by centrifugation, repeatedly washed with ethanol and dried at 60 °C. In the last step, the powder was calcined at 450 °C for 2 hours to obtain pure white TiO_2_. Structural and morphological characterization was done by using a Rigaku Miniflex-II X-ray diffractometer (Cu Kα radiation, λ = 1.5406 Å, 40 kV, 30 mA), a Bruker Vertex 70 FT-IR instrument (16 scans at 4 cm^−1^ resolution), and a Hitachi S-4700 Type II scanning electron microscope (SEM) with 10 kV accelerating voltage equipped with a Röntec QX2 energy dispersive X-ray spectrometer (EDX).

**2.3. Analytical methods**

Mixed liquor suspended solids (MLSS), concentration of mixed liquor volatile suspended solids (MLVSS), and settleability (sludge volume index after five minutes of sedimentation-SVI_5_). These parameters were analyzed following the standard methods (Zheng et al., 2017).

During the extraction of EPS, 20 mL MLSS from the reactors was washed three times with deionized water. After centrifugation (5000 g, 4 °C, 10 min), the supernatant was discarded and the pellet resuspended. The sludge mixture was then heated at 80 °C for 30 min under continuous stirring (200 rpm), and the resulting mixture was centrifuged and its supernatant filtered (0.45 μm) (Li and Yang, 2007). The polysaccharide (PS) and protein (PN) contents in the EPS were assessed using the Anthrone (glucose as the standard) and modified Lowry (bovine serum albumin as the standard) methods (Zhang et al., 2017). The EPS was considered to be the sum of PN and PS. All analyses were performed in triplicate. Granular sludge samples were washed three times with 0.1 M phosphate buffer and then subjected to freeze-drying. The resulting dry granules were examined using scanning electron microscope (SEM) to investigate morphological and structural changes.

**2.4. Determinations of SAUR, SNIUR, SNUR, and SPUR**

The assessment of SAUR, SNIUR and SNUR were performed in a 500 mL Erlenmeyer flask with 50 mL of aerobic granular sludge from the SBR and 350 mL of synthetic wastewater. The sludge and synthetic wastewater in the Erlenmeyer flask were mixed by a magnetic stirrer. The nitrogen sources for the determination of SAUR, SNIUR and SNUR were NH_4_Cl, NaNO_2_, and NaNO_3_ respectively, corresponding to the concentration of 60 mg L^-1^ NH_4_^+^-N, 90 mg L^-1^ NO_2_^‒^-N, and 90 mg L^-1^ NO_3_^‒^-N. The air was introduced into the Erlenmeyer flask by an air diffuser in the determination of SAUR or SNIUR, and the nitrogen gas was filled in the Erlenmeyer flask for anoxic conditions in the SNUR test. The mixed liquor suspended solids (MLSS) in the BOD bottle and Erlenmeyer flask were regarded as an invariant during the determinations of SAUR, SNIUR and SNUR due to the shorter operational time. The SAUR, SNIUR and SNUR were calculated by monitoring the decreased rate of NH_4_-N, NO_2_-N, and NO_3_-N versus time, respectively (Wang et al., 2015).

The specific phosphorus uptake rate (SPUR) was measured according to the methodology suggested by Panswad et al. (2003). The SPUR was determined by taking the sludge at the end of aerobic cycle, washed three times with Milli-Q water and was kept at the appropriate temperatures. The washed sludge was then placed into a 500 mL Erlenmeyer flask equipped with magnetic stirrer for mixing and was incubated at the system temperature. The mixed liquor was dosed with K_2_HPO_4_ and KH_2_PO_4_ to an initial phosphorus concentration of 60 mg L^-1^. The SPUR was calculated by monitoring the decreased rate of phosphorus concentrations as a function of time.

**3. Results and discussion**

**3.1. Characterization of TiO_2_ NPs**

The X-ray diffraction patterns of the TiO_2_ nanocrystals are displayed in *Fig. S1a*. Reflections can be indexed as anatase TiO_2_ (JCPDS card file no. 21-1272), where the peaks appeared at 2θ of 25.2°, 37.8°, 48.1°, 54.6°, 62.6°, 69.7°, and 75.3° correspond to the (101), (004), (200), (211), (204), (220), and (215) crystal planes (John et al., 2020). To obtain more compositional details on TiO_2_, FT-IR measurement was performed, and the resulting spectrum is shown in *Fig. S1b*. The vibrational modes that are commonly attributed to the stretching of Ti–O–Ti of the TiO_2_ nanospheres are observed at 410 and 780 cm^−1^. Furthermore, the vibrational modes at around 1632 and 3420 cm^−1^ are associated with the bending mode of the physisorbed water molecules on TiO_2_, respectively (Eddy et al., 2021). Energy dispersive X-ray (EDX) analysis was carried out to confirm the chemical composition of the as-prepared nanoparticles, and a typical EDX spectrum is shown in *Fig. S1c*. A Ti:O atomic ratio of 1:3 was found from the average measured at three different points of the sample (inset *Fig. S1c*). Scanning electron microscopy image of the TiO_2_ sample is displayed in *Fig. S1d*. The TiO_2_ crystals have spherical morphology of a diameter ranging between 30 and 130 nm.


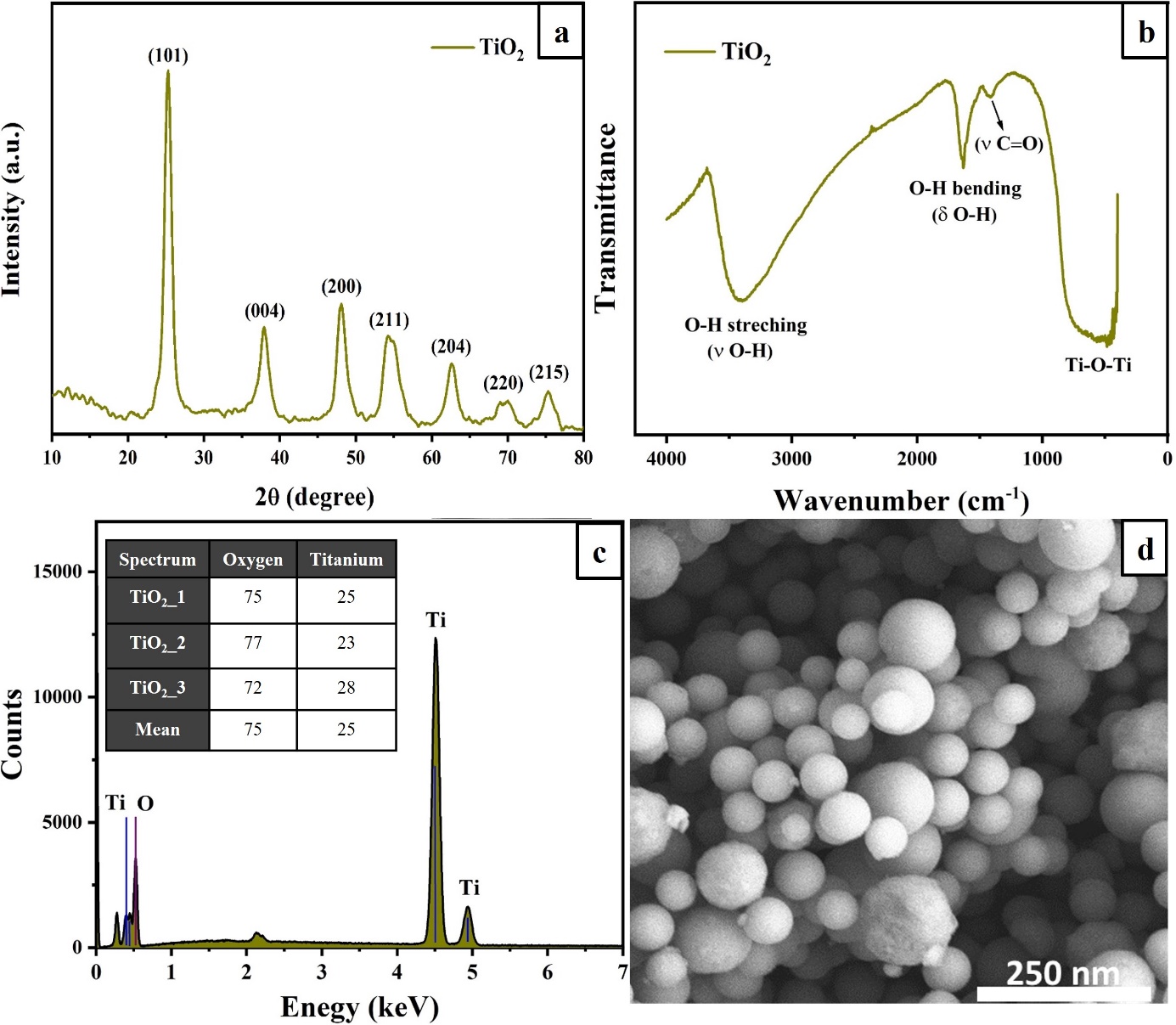


**Fig. S1** Characterization of the TiO_2_ NPs. **a)** X-ray diffraction pattern; The **b)** FT-IR spectrum, **c)** EDX spectrum, and **d)** SEM image of the nanoparticles


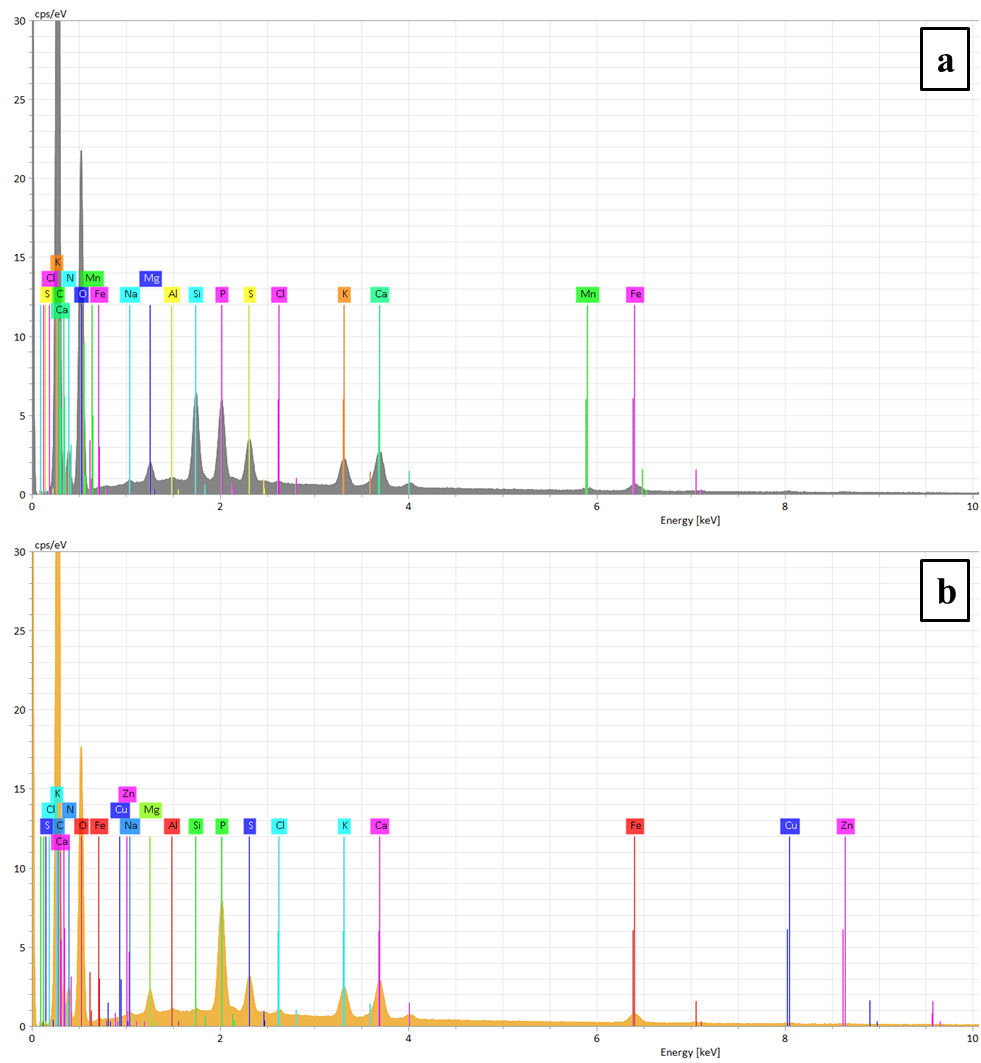


**Fig. S2** EDX spectrum of the **a)** control AGS, and the **b)** control ABGS


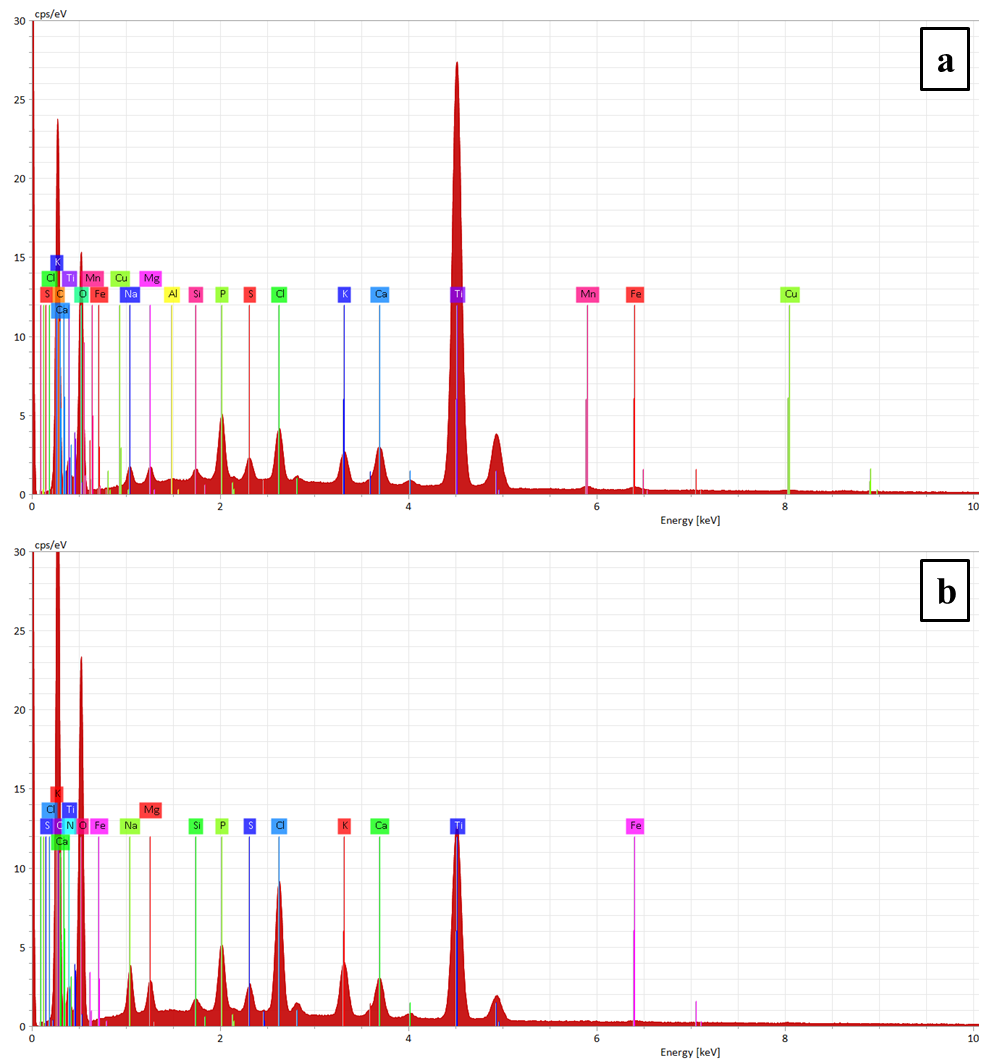


**Fig. S3** EDX spectrum of the **a)** AGS, and the **b)** ABGS after the introduction of 50 mg L^-1^ TiO_2_ NPs

**References**

Eddy, D.R., Ishmah, S.N., Permana, M.D., Firdaus, M.L., Rahayu, I., El-Badry, Y.A., Hussein, E.E., El-Bahy, Z.M., 2021. Photocatalytic phenol degradation by silica-modified titanium dioxide. Appl. Sci. 11. https://doi.org/10.3390/app11199033

John, A.K., Palaty, S., Sharma, S.S., 2020. Greener approach towards the synthesis of titanium dioxide nanostructures with exposed {001} facets for enhanced visible light photodegradation of organic pollutants. J. Mater. Sci. Mater. Electron. 31, 20868–20882. https://doi.org/10.1007/s10854-020-04602-1

Kedves, A., Sánta, L., Balázs, M., Kesserű, P., Kiss, I., Rónavári, A., Kónya, Z., 2020. Chronic responses of aerobic granules to the presence of graphene oxide in sequencing batch reactors. J. Hazard. Mater. 389. https://doi.org/10.1016/j.jhazmat.2019.121905

Li, X.Y., Yang, S.F., 2007. Influence of loosely bound extracellular polymeric substances (EPS) on the flocculation, sedimentation and dewaterability of activated sludge. Water Res. 41, 1022–1030. https://doi.org/https://doi.org/10.1016/j.watres.2006.06.037

Li, Z.Q., Que, Y.P., Mo, L.E., Chen, W.C., Ding, Y., Ma, Y.M., Jiang, L., Hu, L.H., Dai, S.Y., 2015. One-Pot Synthesis of Mesoporous TiO2 Micropheres and Its Application for High-Efficiency Dye-Sensitized Solar Cells. ACS Appl. Mater. Interfaces 7, 10928–10934. https://doi.org/10.1021/acsami.5b02195

Panswad, T., Doungchai, A., Anotai, J., 2003. Temperature effect on microbial community of enhanced biological phosphorus removal system. Water Res. 37, 409–415. https://doi.org/https://doi.org/10.1016/S0043-1354(02)00286-5

Wang, S., Gao, M., Wang, Z., She, Z., Jin, C., Zhao, Y., Guo, L., Chang, Q., 2015. Effect of oxytetracycline on performance and microbial community of an anoxic–aerobic sequencing batch reactor treating mariculture wastewater. RSC Adv. 5, 53893–53904. https://doi.org/10.1039/C5RA06302G

Zhang, B., Ji, M., Wang, F., Li, R., Zhang, K., Yin, X., Li, Q., 2017. Damage of EPS and cell structures and improvement of high-solid anaerobic digestion of sewage sludge by combined (Ca(OH)2 + multiple-transducer ultrasonic) pretreatment. RSC Adv. 7, 22706–22714. https://doi.org/10.1039/c7ra01060e

Zheng, X.Y., Lu, D., Chen, W., Gao, Y.J., Zhou, G., Zhang, Y., Zhou, X., Jin, M.Q., 2017. Response of Aerobic Granular Sludge to the Long-Term Presence of CuO NPs in A/O/A SBRs: Nitrogen and Phosphorus Removal, Enzymatic Activity, and the Microbial Community. Environ. Sci. Technol. 51, 10503–10510. https://doi.org/10.1021/acs.est.7b02768
